# Social factors associated with self-reported changes in mental health symptoms among youth in the COVID-19 pandemic: a cross-sectional survey

**DOI:** 10.1186/s12889-024-18087-8

**Published:** 2024-02-28

**Authors:** Stephana Julia Moss, Maia Stelfox, Eric McArthur, Cynthia Sriskandarajah, Sofia B. Ahmed, Kathryn Birnie, Donna M. Halperin, Scott A. Halperin, Micaela Harley, Jia Hu, Josh Ng Kamstra, Laura Leppan, Angie Nickel, Nicole Racine, Kristine Russell, Stacie Smith, May Solis, Perri R. Tutelman, Henry T. Stelfox, Kirsten M. Fiest, Jeanna Parsons Leigh

**Affiliations:** 1 Faculty of Health, Dalhousie University, Halifax, NS Canada; 2 London Health Sciences Centre, London, ON UK; 3 Cumming School of Medicine, University of Calgary, Calgary, AB Canada; 4 Departments of Anesthesiology, Perioperative, and Pain Medicine, Calgary, AB Canada; 5 Rankin School of Nursing, St. Francis Xavier University, Antigonish, NS Canada; 6 Canadian Center for Vaccinology, Departments of Pediatrics and Microbiology & Immunology, Dalhousie University, Halifax, NS Canada; 7 Frayme, Cornwall, ON Canada; 8 Department of Community Health Sciences, University of Calgary, Calgary, AB Canada; 9 Department of Surgery, Massachusetts General Hospital and Harvard Medical School, Boston, MA USA; 10 Faculty of Social Sciences, University of Ottawa, Children’s Hospital of Eastern Ontario Research Institute, Ottawa, ON Canada; 11 Young Canadian Roundtable On Health, Toronto, ON Canada; 12 Department of Oncology, University of Calgary, Calgary, AB Canada; 13 Faculty of Medicine and Dentistry, University of Alberta, Edmonton, AB Canada; 14 Department of Critical Care Medicine and O’Brien Institute for Public Health, University of Calgary, Calgary, AB Canada

**Keywords:** Child, Youth, COVID-19, Mental Health, Sociodemographics

## Abstract

**Background:**

Children and youth experienced marked impacts on day-to-day life in the COVID-19 pandemic that were associated with poorer familial and friend relationships, and greater mental health challenges. Few studies provide self-report data on mental health symptoms from children and youth themselves. We sought to examine the associations between social factors and child and youth self-reported symptoms of worsened mood, anxiety, and irritability during the COVID-19 pandemic.

**Methods:**

A nationally representative cross-sectional survey was administered online to collect self-report data across 10 Canadian provinces among children (11–14 years) and youth (15–18 years), April–May 2022. Age-appropriate questions were based on The Partnership for Maternal, Newborn & Child Health and the World Health Organization of the United Nations H6 + Technical Working Group on Adolescent Health and Well-Being consensus framework and the Coronavirus Health and Impact Survey. Associations between a priori defined social factors (e.g., relationship quality) and respondent self-reported mental health were evaluated using ordinal logistic regression models adjusted for age, sex, and geographic location.

**Results:**

We analyzed data from 483 (51.7%) children (11–14 years; 227, 47.0% girls) and 450 (48.3%) youth (15–18 years; 204, 45.3% girls). The parents of most children and youth had resided in Canada for over 20 years (678, 72.7%). Over one-quarter of children and youth self-identified as Black, Indigenous, or a Person of Color (134, 27.7%; 134, 29.8%, respectively). Over one-third of children and youth self-reported symptoms of worsened mood (149, 30.9%; 125, 27.8%, respectively), anxiety (181, 37.5%; 167, 37.1%, respectively), or irritability (160, 33.1%; 160, 35.6%, respectively) during, compared to pre-pandemic. In descending order of odds ratios (OR), for children and youth, worsened familial relationships (during compared to pre-pandemic) was associated with the self-reported symptoms of worsened mood (child: OR 4.22, 95%CI 2.51–6.88; youth: OR 6.65 95%CI 3.98–11.23), anxiety (child: OR 4.24, 95%CI2.69–6.75; youth: OR 5.28, 95%CI 3.17–8.86), and irritability (child: OR 2.83, 95%CI 1.76–4.56; youth: OR 6.46, 95%CI 3.88–10.90).

**Conclusions:**

Self-reported data from a nationally representative sample of children and youth suggest strong associations between social factors and mental health during the COVID-19 pandemic. Interventions targeting child and youth familial relationships may positively impact child and youth mental health.

**Supplementary Information:**

The online version contains supplementary material available at 10.1186/s12889-024-18087-8.

## Introduction

Children and youth have experienced mental health problems with greater frequency since the onset of the COVID-19 pandemic [1]. Across 29 studies, including 80,879 youth globally, the pooled prevalence estimates of child and youth depression and anxiety were 25.2% and 20.5%, respectively, having doubled compared to pre-pandemic estimates [2]. Other studies have reported increases in child and youth externalizing behaviors (e.g., hyperactivity and conduct problems) [3]. Lockdowns and school closures, in addition to disruptions to daily routines, have contributed to this increase in mental health difficulties among children and youth [4, 5], though most published studies have reported data from parents of children and youth while few provided self-report data from a nationally representative sample of children and youth themselves.

Especially among lower-income and less-educated families, as well as families from ethnic minority and vulnerable groups, pandemic-related disruptions coincided with negative impacts to social factors such as familial and friend relationships, and support for mental and physical wellbeing [6, 7]. Elevated symptoms of depression and anxiety in mothers of young children nearly doubled and tripled, respectively, from before the pandemic [2]. Increases in parental mental health problems during the COVID-19 pandemic impacted parenting behaviors and parent–child relationships; greater parental psychological distress was associated with a higher likelihood of engaging in more negative and fewer positive parenting practices during the pandemic [8]. Higher levels of depression in mothers and fathers were related to greater parent–child relationship conflict [9]. Pandemic stressors also had broader implications for entire social networks of children and youth, linked to poorer friendship quality which, in turn, was associated with greater mental health challenges [10, 11].

Extensive prior research on large-scale health, economic, and sociopolitical crises report short-and long-term health consequences for children, youth, and families. For example, studies conducted within pandemics prior to the COVID-19 pandemic (e.g., SARS, H1N1) documented inadequate adjustment (e.g., anxiety, depression, posttraumatic stress disorder) among children and youth who directly experienced pandemic-related stressors [12–14]. Similarly, momentous economic downturns, such as the Great Recession of 2008, have been linked to challenges with child and youth behavior problems, self-efficacy, and school attendance [15–17]. Within the COVID-19 pandemic, studies on family functioning found that individual emotions were influential amongst families with emotion contagion negatively impacting family regulation [18], while others reported large deteriorations in familial mental and behavioral health [19].

We previously conducted a large, nationally representative, multi-informant cross-sectional survey to report how mental health symptoms of children, youth, and their parents changed during COVID-19 compared to pre-pandemic times [20]. In that study, we found that children and youth were significantly less likely to self-report worsened mental health symptoms compared to their parents, and that children and youth most frequently self-reported symptoms of worsened mood, anxiety, and irritability. The objective of the present analytical study was to use child and youth self-report data from our cross-sectional survey to identify social factors associated with self-report symptoms of worsened mood, anxiety, and irritability among children and youth during the COVID-19 pandemic. Better understanding on the association of social factors with child and youth mental health provides the opportunity to reimagine how public mental health interventions are developed, tested, and implemented. Careful consideration of the aims and population-level impacts of child and youth mental health interventions complemented by assessment of interpersonal and environmental elements may facilitate more effective study of child and youth mental health during and after a public health crisis that does not shy away from complexity.

## Methods

### Study design and population

Data were collected from an anonymous, voluntary, 10-min cross-sectional survey administered by Leger, a Canadian-based market research and polling firm (https://leger360.com), between April 20, 2022, and May 25, 2022 (the time period that Canadian provinces had eased public health measures such as physical distancing and masking requirements) [21]. Leger uses a dynamic Leger Opinion (LEO) panel that is an online pool of over 400,000 individuals recruited and validated through multiple methods. Participants in the LEO panel consented to be contacted for research purposes and at any given time, reflect a representative sample of Canadian residents with internet access. Respondents to our survey received LEO reward points after completing the questionnaire that could be redeemed for gift cards and merchandise. Assuming children and youth aged 11–18 represent ~ 11% (~ 4 million) [22] of the Canadian population, we recruited 1600 respondents (800 parent–child/youth dyads (i.e., a group of two members)) to conduct subgroup analyses with a ± 3.5% margin of error at a 95% confidence level (95% CI). At least 15% of the sample was required to be dyads who had lived in Canada for fewer than 10 years; 5% of the sample was required to be dyads who had lived in Canada for less than 5 years. The (total) 85-item (English and French) electronic survey was administered to LEO panelists who identified as parents or legal guardians (> 18 years of age; hereafter referred to as parents) with at least one child (11–14 years of age) or youth (15–18 years of age) living in the same household; the oldest child or youth was selected if more than one was eligible. Age ranges for children and youth were selected to align with Statistics Canada standards and to adhere to institutional ethical requirements (e.g., age-tailored questions) [23]. Parents were asked the first 45-items, and their child or youth were asked the subsequent 40-items. We followed the Checklist for Reporting Results of Internet E-Surveys (CHERRIES) guidelines (Supplemental Table 1) [24].

**Table 1:** Demographics and characteristics of 933 child and youth participants

Characteristic	Child (11–14 y) Value, No. (%) *N* = 483	Youth (15–18 y) Value, No. (%) *N* = 450
Sex^a^		
Male	239 (49.5)	235 (52.2)
Female	239 (49.5)	214 (47.6)
Prefer not to answer	5 (1.0)	1 (0.2)
Gender^b^		
Woman	227 (47.0)	204 (45.3)
Man	242 (50.1)	226 (50.2)
Non-binary	2 (0.4)	6 (1.3)
Two-Spirit	1 (0.1)	2 (0.4)
Prefer not to answer	2 (0.4)	7 (1.6)
Disability		
Yes – visible	11 (2.3)	11 (2.4)
Yes – invisible	31 (6.4)	23 (5.1)
No	439 (90.9)	414 (92.0)
Ethnicity^c^		
Black, Indigenous, and People of color	134 (27.7)	134 (29.8)
White	335 (69.4)	317 (70.4)
Prefer to self-describe	14 (2.9)	5 (0.01)
Self-rated COVID-19 knowledge		
Very poor	19 (3.9)	12 (2.7)
Poor	62 (12.8)	64 (14.2)
Average	252 (52.2)	210 (46.7)
Good	129 (26.7)	111 (24.7)
Very good	21 (4.4)	52 (11.6)
Previously diagnosed with COVID-19		
Yes	172 (35.6)	141 (31.3)
No	310 (64.2)	306 (68.0)
Job loss during COVID-19 pandemic		
Yes		114 (25.3)
No		138 (30.7)
Not applicable	483 (100.0)	198 (44.0)
Social media use per day, hours		
None	99 (20.5)	23 (5.1)
< 1	122 (25.3)	95 (21.1)
1–3	155 (32.1)	198 (44.0)
4–6	83 (17.2)	90 (20.0)
> 6	24 (5.0)	44 (9.8)

^a^Missing 5 responses for children, and 1 response for youth

^b^Missing 2 response for children, and 7 responses for youth

^c^Missing 14 responses for children, and 5 responses for youth

### Survey development

We created a preliminary list of social health and mental health questions based on findings presented in published articles identified in our scoping review [25] and systematic review [26] on strategies, approaches, and interventions targeted to improve youth wellbeing during the COVID-19 pandemic. Preliminary questions were mapped onto The Partnership for Maternal, Newborn & Child Health and the World Health Organization of the United Nations H6 + Technical Working Group on Adolescent Health and Well-Being consensus framework for defining, programming, and measuring adolescent wellbeing that is part of a broader program of work that includes a multi-user Call to Action to prioritize adolescent well-being [27]. This framework includes five domains: (1) Good health and optimum nutrition; (2) Connectedness, positive values, and contribution to society; (3) Safety and a supportive environment; (4) Learning, competence, education, skills, and employability; and (5) Agency and resilience (Supplemental Table 2). Demographic questions were based on the Coronavirus Health and Impact Survey (CRISIS) [28]. We developed a combination of continuous, categorial, Likert-type, and open-ended response options; Likert-type questions included a scale ranging from 1 (i.e., “a little”) to 5 (i.e., “a lot”). Questions were iteratively refined by the core survey development team (JPL, SJM, RBM, DMH, SAH, PT) [29] and six public citizen partners (three youth: MS, MH, SS, and three parents: KR, MS, AN). The order of the response options was randomized, and attention checks (i.e., innocuous questions with a single correct answer) were randomly inserted throughout the questionnaire. One question was presented per screen and respondents were able to change their answer once they moved to the next screen; all questions included a “don’t know” or “prefer not to answer” option that were excluded from analyses.

### Outcomes

In our earlier work that reported on a multi-informant cross-sectional survey, we identified that children and youth most frequently self-reported symptoms of worsened mood, anxiety, and irritability [20]; these three variables were selected as primary outcomes for the present study. Respondents (children and youth) were asked, “Compared to the time before the COVID-19 pandemic, how is your [mood/anxiety/irritability]”. Operational, age-appropriate definitions for mental health symptoms provided to the participant at the time of survey conduction were as follows: 1) Mood (*Parent and Youth*: Poor mood might be when you feel empty, helpless, or inadequate, have low self-esteem or loss of interest in usual activities; *Child*: Feeling sad or down or not wanting to do the things that you like to do); 2) Anxiety (*Parent and Youth*: An emotion characterized by feelings of tension, worried thoughts, and physical changes like increased blood pressure; *Child*: Having thoughts or feelings that can be very scary or that worry you); and 3) Irritability (*Parent and Youth*: Irritability involves feelings of anger or frustration that often arise over even the smallest of things; *Child*: Being annoyed easily at things going on around you).

### Social factors

Social factors were selected a priori based on findings from our scoping review [25], systematic review [26], and cross-sectional survey [20] in this research area. Social factors included self-reported “good” pre-pandemic physical health, mental health and nutrition (disagree versus neutral or agree), “good” physical health support and mental health support (disagree versus neutral or agree), distress related to school closures (extremely/very versus moderately/slightly/not at all), family and friend relations (worse versus about the same or better), sleep quantity (< 8 h versus 8 + hours per night), exercise quantity (< 3 days of 30 min (of organized sports and free play, cumulatively) versus 3 + days of 30 min (of organized sports and free play, cumulatively) per week), and social media quantity (4 + hours versus < 4 h per day).

### Statistical analysis

Continuous variables were summarized using mean (standard deviation, SD) or median (interquartile range, IQR). Categorical variables were presented as frequency (percentage). Survey question responses were evaluated separately for children (11–14 years old) and youth (15–18 years old). Potential associations between a priori defined factors and respondent mental health outcomes (i.e., mood, anxiety, irritability) were evaluated using ordinal logistic regression models and reported as odds ratios with 95% confidence intervals for the odds of worsened mental health. Adjusted models included age (continuously, per year), sex (female as referent), and geographic location (Central, Atlantic, Prairie, British Columbia, with Central as referent). No missing data were present for any social factor or outcome variable. As these analyses were considered exploratory and hypothesis-generating, we did not adjust the statistical significance level for multiple testing; the width of reported confidence intervals may not be used in place of hypothesis testing as they have not been adjusted for multiplicity. All analyses were conducted using R version 4.2.1 [30].

### Patient and public involvement

We abided by the Canadian Institutes of Health Research (CIHR)-guiding core principles of inclusiveness, mutual respect, support, and co-building [31] and adhered to the GRIPP-2 reporting guidelines for patient and public involvement [32]. Youth and parent involvement in the current project began in 2021; they participated in group discussions alongside other stakeholders (e.g., researchers, clinicians, decision makers). The research questions, protocol, and this paper were jointly developed with youth (MS, SS, MH) and parent (AN, MS, KR) partners on this team. All youth and family partners were compensated for their time.

### Ethical considerations

All participants provided electronic informed consent on their own behalf; as the parent had significant knowledge of their child/youth, prior to submitting their own consent, the parent attested that they understood the information regarding their child/youth’s participation and that their child/youth had the capacity to consent on their own behalf. This study was approved by the University of Calgary’s Conjoint Health Research Ethics Board (#21–2013) and the Research Ethics Board at Dalhousie University (#2021–5947); all methods were carried out according to research ethics board guidelines and regulations.

## Results

### Survey participants

We surveyed 483 (51.7%) children (aged 11–14 years; *n* = 227, 47.0% girls), and 450 (48.3%) youth (aged 15–18 years; *n* = 204, 45.3% girls) (Table 1; Supplemental Table 2). Children and youth were most commonly from households comprised of four or fewer members (*n* = 698, 74.8%), and most families had resided in Canada for over 20 years (*n* = 678, 72.7%). Over one-quarter of children and youth self-identified as Black, Indigenous, or a Person of Color (*n* = 134, 27.7%; *n* = 134, 29.8%, respectively).

### Mental health outcomes

**Fig. 1:**
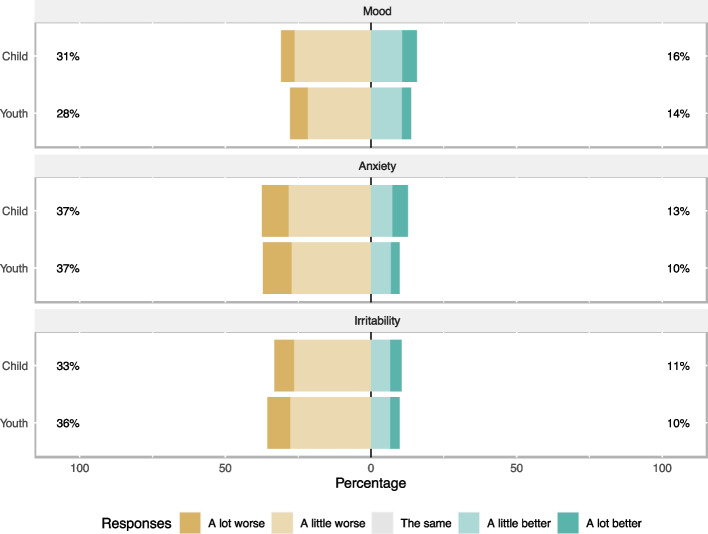
Mental health impacts of 933 child/youth survey participants. Respondents were asked, Compared to the time before the COVID-19 pandemic, how is your [mental health domain]

Children and youth in our sample self-reported symptoms of worsened mood (*n* = 149, 30.9%; *n* = 125, 27.8%, respectively), anxiety (*n* = 181, 37.5%; *n* = 167, 37.1%, respectively), or irritability (*n* = 160, 33.1%; *n* = 160, 35.6%, respectively) (Fig. 1).

#### Mood

Children who experienced worsened familial relationships during compared to pre-pandemic (OR 4.22, 95%CI 2.61–6.88), worsened relationships with friends during compared to pre-pandemic (OR 2.41, 95%CI 1.62–3.61), or stress regarding school closures during the COVID-19 pandemic (OR 1.72, 95%CI 1.19–2.48), were more likely to self-report symptoms of worsened mood (Table 2). Children who exercised for 30 min on fewer than three days per week during COVID-19 were also more likely to self-report symptoms of decreased mood (OR 1.65, 95%CI 1.16–2.36). The potential predictors for symptoms of worsened mood among youth in our sample were worsened familial relationships during compared to pre-pandemic (OR 6.65, 95%CI 3.98–11.23), worsened relationships with friends during compared to pre-pandemic (OR 3.33, 95%CI 2.12–5.26), and stress regarding school closures during the COVID-19 pandemic (OR 2.24, 95%CI 1.52–3.32) (Table 3). Among youth poor pre-pandemic mental health (OR 1.56, 95%CI 1.02–2.39) and poor pre-pandemic mental health support (OR 1.57, 95%CI 1.00–2.45) were associated with symptoms of decreased mood.

**Table 2:**
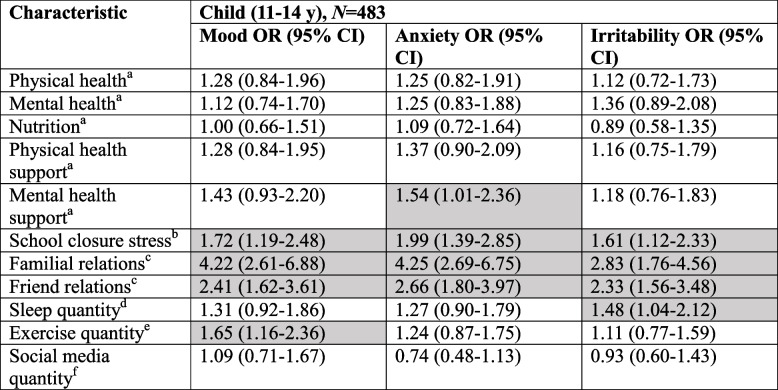
Adjusted multivariate analyses for child (11-14 years) mental health symptoms

Shaded cells are statistically significant

Adjusted models included age (continuously, per year), sex (female as referent), and geographic location (Central, Atlantic, Prairie, BC, with Central as referent)

*Abbreviations*: *95% CI* 95% Confidence Interval

^a^Disagree versus Neutral or Agree [Prior to the COVID-19 pandemic, I had good…]

^b^Extremely/Very versus Moderately/Slightly/Not at all [How stressful have school closures been for you?]

^c^Worse versus About the same or better [How has the quality of your relations changed?]

^d^ < 8 h versus 8 + hours (per night) [During the past two weeks, on average, how many hours did you sleep on weekdays?]

^e^ < 3 days versus 3 + days (per week) [During the past two weeks, how many days per week did you exercise for at least 30 min?]

^f^4 + hours versus < 4 h (per day) [During the past two weeks, how much time did you spend using social media?]

**Table 3:**
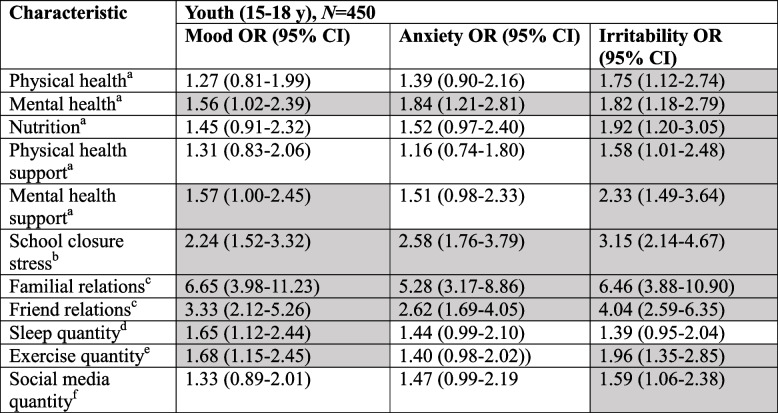
Adjusted multivariate analyses for youth (15-18 years) mental health symptoms

Shaded cells are statistically significant

Adjusted models included age (continuously, per year), sex (female as referent), and geographic location (Central, Atlantic, Prairie, BC, with Central as referent)

*Abbreviations*: *95% CI* 95% Confidence Interval

^a^isagree versus Neutral or Agree [Prior to the COVID-19 pandemic, I had good…]

^b^Extremely/Very versus Moderately/Slightly/Not at all [How stressful have school closures been for you?]

^c^Worse versus About the same or better [How has the quality of your relations changed?]

^d^ < 8 h versus 8 + hours (per night) [During the past two weeks, on average, how many hours did you sleep on weekdays?]

^e^ < 3 days versus 3 + days (per week) [During the past two weeks, how many days per week did you exercise for at least 30 min?]

^f^4 + hours versus < 4 h (per day) [During the past two weeks, how much time did you spend using social media?]

#### Anxiety

Worsened familial relationships during compared to pre-pandemic (OR 4.25, 95%CI 2.69–6.75), worsened relationships with friends during compared to pre-pandemic (OR 2.66, 95%CI 1.80–3.97), and stress related to school closures during the COVID-19 pandemic (OR 1.99, 95%CI 1.39–2.85) were associated with self-report symptoms of worsened anxiety among children (Table 2). Poor pre-pandemic mental health support—though not pre-pandemic poor mental health itself—was also associated with self-report symptoms of worsened anxiety for children (OR 1.54, 95%CI 1.01–2.36). Among youth, worsened familial relationships during compared to pre-pandemic (OR 5.28, 95%CI 3.17–8.86), worsened relationships with friends during compared to pre-pandemic (OR 2.62, 95%CI 1.69–4.05), and stress related to school closures during the COVID-19 pandemic (OR 2.58, 95%CI 1.76–3.79) were associated with self-report symptoms of worsened anxiety; poor pre-pandemic mental health (OR 1.84, 95%CI 1.21–2.81) was also associated with self-report symptoms of worsened anxiety, but poor pre-pandemic mental health support was not (Table 3).

#### Irritability

Children who experienced worsened familial relationships during compared to pre-pandemic (OR 2.83, 95%CI 1.76–4.56), worsened relationships with friends during compared to pre-pandemic (OR 2.33, 95%CI 1.56–3.48), stress related to school closures during the COVID-19 pandemic (OR 1.61, 95%CI 1.12–2.33), as well as those who on average slept less than eight hours per night during the COVID-19 pandemic (OR 1.48, 95%CI 1.04–2.12) were more likely to self-report symptoms of worsened irritability (Table 2). Among youth, every social factor tested but for sleep quantity was found to be associated with self-report symptoms of worsened irritability, with potential predictors being worsened familial relationships during compared to pre-pandemic (OR 6.46, 95%CI 3.88–10.90), worsened relationships with friends during compared to pre-pandemic (OR 4.04, 95%CI 2.59–6.35), and stress related to school closures during the COVID-19 pandemic (OR 3.15, 95%CI 2.14–4.67; Table 3).

## Discussion

We conducted a nationally representative survey to collect self-report data on mental health symptoms from diverse children and youth in Canada. This study builds on existing cross-sectional evidence by demonstrating that over one-third of children and youth in our sample self-reported symptoms of worsened mental health during compared to pre-COVID-19 pandemic. Children and youth who reported experiencing changes in familial relationships or relationships with friends or were stressed regarding school closures were more likely to self-report symptoms of worsened mental health. The data highlights the importance of psychosocial interventions grounded in familial relationships that includes strategies to support mental health as well as broader existential concerns and uncertainties regarding personal goals and agency. Our findings overall identify potential social factors that could serve as opportunities for intervention.

Our analyses revealed consistent links between family relationships and child and youth mental health. Family systems research emphasizes the importance of supportive and nurturing parenting for child and youth emotional states [33, 34]. Child/youth-parent conflict is reported to decrease when children and youth use parental social supports in times of crisis [35, 36]. Grounded and stable parental warmth may, therefore act as a protective factor against negative affect and distress among children and youth that arise from curtailed family functioning [37, 38]. Accessible approaches targeting the interparental relationship (with or without a direct parenting focus) can significantly improve couple communication, relationship satisfaction, parenting quality and comprehensiveness, and adult mental health, with associated improved outcomes for child and youth mental health in the family [39–43]. When considering interventions to recover from the COVID-19 pandemic and prevention strategies to prepare for future public health crises, targeting the entire family unit may have instrumental downstream impacts on child and youth mental health [44].

The available literature indicates that not all families were impacted similarly by social disruptions caused by the COVID-19 pandemic [45]. For example, mothers [46], members of racialized groups [47], financially insecure families [48], and those with preexisting mental and/or physical health conditions or belonging to sexually marginalized groups [49], experienced heightened stress due to public health measures to contain COVID-19 [50]. While families who experienced more cumulative stress pre- and/or during the COVID-19 pandemic were more likely to experience worsened mental health outcomes, it is important to consider that risk is a probabilistic—not deterministic— process [51, 52]. Specifically, risk operates in tandem with promotive (that place family members on a trajectory toward positive development and functioning regardless of risk level [53, 54]) and protective (for positive adaptation and functioning when risk or adversity is heightened, over and above any effects at lower risk levels [55]) factors. The mental health effects of the pandemic on families depended primarily on child/youth developmental stages, the type and severity of challenges experienced, preexisting vulnerabilities and strengths, availability (or lack) of resources, and mobilization of protective systems that foster resilience [56].

The COVID-19 pandemic highlighted globally the integral role of educators in child and youth day-to-day lives and wellbeing [57–59]. Personalized parental training that focuses on problem solving and strengthening families within the education system may be a broad and effective intervention for individualized child and youth mental health [60, 61]. Governmental investments and involvement from Ministers of Education to develop and test interventions focused on strengthening the family environment is an attractive approach to educate parents on optimal family functioning strategies [62, 63]. Potential interventions may include key collaborative “check points” among parents and teachers on plans for providing accessible mental health resources to children or youth displaying early signs of mental health challenges [64, 65].

Our data highlights that changes to friend groups and connectedness in the COVID-19 pandemic was an unintended consequence of disease containment measures that were particularly problematic for children and youth [66–68] who often rely on their peer group for personal identity and support during early developmental stages [69, 70]. The propensity to lose connectedness may have exacerbated some of the mental health impacts of disease containment measures [71]. Losing links to friends and feeling excluded can result in a worsened mood [72]. Social anxiety—triggered by a perceived threat to social relationships or status—is also strongly associated with connectedness [73, 74]. Clinically, particularly in periods of substantial social disruption, it is important to encourage parents to establish and maintain structured routines centered on inclusive group activities (virtually, or otherwise) and involving children and youth in creating family social events that foster healthy connection [75, 76]. For a better understanding of the duration, intensity and nature (e.g., peer-related vs. parent-related) of connectedness, measures such as The Social Connectedness Scale or the Social Assurance Scale should be used in the future when evaluating the experiences of children and youth [77].

### Limitations

Our findings highlight the complexity of the relationships between social factors and child and youth mental health symptoms during the COVID-19 pandemic within the context of a high-income country. Research on impacts among low- and middle-income countries has showed comparably elevated prevalence of worsened mental health symptoms, augmented by social, economic and cultural factors, particularly poor access to mental health support [78]. Future research should focus on provision of appropriate mental health support to address systemic inequalities and social determinants to meet child and youth mental health needs in these regions of the world. Further, our data were collected from a large and representative sample of the Canadian population in a cross-sectional survey. However, our results have limited longitudinal applicability; we queried children and youth to self-report retrospectively on perceived changes in mental health symptoms throughout the COVID-19 pandemic and cannot generalize our findings to any additional evolution in mental health symptoms that may have occurred in the post-pandemic period. Additional research that uses longitudinal designs with ideally more than three time points are needed to assess nonlinear change and developmental cascades among children and youth. As age was not one of our a priori defined social factors, we did not seek to understand the association of age with mental health outcomes, despite that age plays as much of a role in psychopathology compared to social and genetic factors. Future studies on this topic should seek to better understand the moderating role of age on mental health outcomes during public health crises. Our survey was deployed online in English and French—Canada’s two official languages—and excluded children and youth without internet access or those who read and write exclusively in other languages (~ 9% and ~ 2% of the Canadian population, respectively) [79]. Utilizing a volunteer panel (Leger’s LEO panel) to recruit children and youth for compensation may also have introduced recruitment bias. The majority of our sample was White, primarily from Central Canada (including Ontario and Quebec); thus, our results should be generalized with caution to children and youth of diverse ethnic backgrounds or those residing in other Canadian provinces.

## Conclusions

Many children and youth experienced symptoms of worsened mental health during the COVID-19 pandemic that were related to social factors, including changes to relationships with family and friends and stress related to school closures. We provide self-report data from a nationally representative sample of children and youth that highlights complex pathways linking social factors with child and youth mental health in the COVID-19 pandemic. When considering interventions to recover from the COVID-19 pandemic and prevention strategies to prepare for future public health crises, targeting child and youth familial relationships may positive impacts on child and youth mental health.

### Supplementary Information

**Supplementary Material 1. **

## Data Availability

The data are not publicly available due to containing semi-identifiable information that could compromise participant privacy. Additional summary tables are available from the corresponding author upon request.
