# Supplementary material for: Social factors associated with self-reported changes in mental health symptoms among youth in the COVID-19 pandemic: a cross-sectional survey
Source: BMC Public Health. 2024 Feb 28;24:631. doi: 10.1186/s12889-024-18087-8 (PMC10900679; doi:10.1186/s12889-024-18087-8)
Supplement: Supplementary file 1 — Supplementary Material 1. [file 12889_2024_18087_MOESM1_ESM.docx]

**Supplemental Table 1.** CHERRIES

| ***Item Category*** | ***Checklist Item*** | ***Explanation*** | ***Page*** |
| --- | --- | --- | --- |
| **Design** | | | |
|  | *Describe survey design* | Describe target population, sample frame. Is the sample a convenience sample? | 8 |
| **IRB (Institutional Review Board) approval and informed consent process** | | | |
|  | *IRB approval* | Mention whether the study has been approved by an IRB. | 9 |
|  | *Informed consent* | Describe the informed consent process. Where were the participants told the length of time of the survey, which data were stored and where and for how long, who the investigator was, and the purpose of the study? | 9 |
|  | *Data protection* | If any personal information was collected or stored, describe what mechanisms were used to protect unauthorized access. | 8 |
| **Development and pre-testing** | | | |
|  | *Development and testing* | State how the survey was developed, including whether the usability and technical functionality of the electronic questionnaire had been tested before fielding the questionnaire. | 8 |
| **Recruitment process and description of the sample having access to the questionnaire** | | | |
|  | *Open survey versus closed survey* | An “open survey” is a survey open for each visitor of a site, while a closed survey is only open to a sample which the investigator knows (password-protected survey). | 9 |
|  | *Contact mode* | Indicate whether or not the initial contact with the potential participants was made on the Internet. (Investigators may also send out questionnaires by mail and allow for Web-based data entry.) | 9 |
|  | *Advertising the survey* | How/where was the survey announced or advertised? Some examples are offline media (newspapers), or online (mailing lists – If yes, which ones?) or banner ads (Where were these banner ads posted and what did they look like?). It is important to know the wording of the announcement as it will heavily influence who chooses to participate. Ideally the survey announcement should be published as an appendix. | 9 |
| **Survey administration** | | | |
|  | *Web/E-mail* | State the type of e-survey (eg, sent out through e-mail). If it is an e-mail survey, were the responses entered manually into a database, or was there an automatic method for capturing responses? | 9 |
|  | *Context* | Describe the Web site (for mailing list/newsgroup) in which the survey was posted. What is the Web site about, who is visiting it, what are visitors normally looking for? Discuss to what degree the content of the Web site could pre-select the sample or influence the results. | 9 |
|  | *Mandatory/voluntary* | Was it a mandatory survey to be filled in by every visitor who wanted to enter the Web site, or was it a voluntary survey? | 9 |
|  | *Incentives* | Were any incentives offered (eg, monetary, or non-monetary incentives? | 8 |
|  | *Time/Date* | In what timeframe were the data collected? | 8 |
|  | *Randomization of items or questionnaires* | To prevent biases items can be randomized or alternated. | 9 |
|  | *Adaptive questioning* | Use adaptive questioning (certain items, or only conditionally displayed based on responses to other items) to reduce number and complexity of the questions. | 9 |
|  | *Number of Items* | What was the number of questionnaire items per page? | 8 |
|  | *Completeness check* | It is technically possible to do consistency or completeness checks before the questionnaire is submitted. Was this done, and if “yes”, how? An alternative is to check for completeness after the questionnaire has been submitted. If this has been done, it should be reported. All items should provide a non-response option such as “not applicable” or “rather not say”, and selection of one response option should be enforced. | 9 |
|  | *Review step* | State whether respondents were able to review and change their answers (eg, through a Back button or a Review step which displays a summary of the responses and asks the respondents if they are correct). | 9 |
| **Response rates** | | | |
|  | *Unique site visitor* | If you provide view rates or participation rates, you need to define how you determined a unique visitor. There are different techniques available, based on IP addresses or cookies or both. | N/A |
|  | *View rate (Ratio of unique survey visitors/unique site visitors)* | Requires counting unique visitors to the first page of the survey, divided by the number of unique site visitors (not page views!). It is not unusual to have view rates of less than 0.1 % if the survey is voluntary. | N/A |
|  | *Participation rate (Ratio of unique visitors who agreed to participate/unique first survey page visitors)* | Count the unique number of people who filled in the first survey page (or agreed to participate, for example by checking a checkbox), divided by visitors who visit the first page of the survey (or the informed consents page, if present). This can also be called “recruitment” rate. | N/A |
|  | *Completion rate (Ratio of users who finished the survey/users who agreed to participate)* | The number of people submitting the last questionnaire page, divided by the number of people who agreed to participate (or submitted the first survey page). This is only relevant if there is a separate “informed consent” page or if the survey goes over several pages. This is a measure for attrition. Note that “completion” can involve leaving questionnaire items blank. This is not a measure for how completely questionnaires were filled in. (If you need a measure for this, use the word “completeness rate”.) | 9 |
| **Preventing multiple entries from the same individual** | | | |
|  | *Cookies used* | Indicate whether cookies were used to assign a unique user identifier to each client computer. If so, mention the page on which the cookie was set and read, and how long the cookie was valid. Were duplicate entries avoided by preventing users access to the survey twice; or were duplicate database entries having the same user ID eliminated before analysis? In the latter case, which entries were kept for analysis (eg, first or the most recent)? | 8, 9 |
|  | *IP check* | Indicate whether the IP address of the client computer was used to identify potential duplicate entries from the same user. If so, mention the period of time for which no two entries from the same IP address were allowed (eg, 24 hours). Were duplicate entries avoided by preventing users with the same IP address access to the survey twice; or were duplicate database entries having the same IP address within a given period of time eliminated before analysis? If the latter, which entries were kept for analysis (eg, the first entry or the most recent)? | N/A |
|  | *Registration* | In “closed” (non-open) surveys, users need to login first and it is easier to prevent duplicate entries from the same user. Describe how this was done. For example, was the survey never displayed a second time once the user had filled it in, or was the username stored together with the survey results and later eliminated? If the latter, which entries were kept for analysis (eg, the first entry or the most recent)? | 8, 9 |
| **Analysis** | | | |
|  | *Handling of incomplete questionnaires* | Were only completed questionnaires analyzed? Were questionnaires which terminated early (where, for example, users did not go through all questionnaire pages) also analyzed? | 10 |
|  | *Statistical correction* | Indicate whether any methods such as weighting of items or propensity scores have been used to adjust for the non-representative sample; if so, please describe the methods. | 10 |

**Supplemental Table 2.** The Partnership for Maternal, Newborn & Child Health and the World Health Organization of the United Nations H6+ Technical Working Group on Adolescent Health and Well-Being consensus framework^1^

| **Domain** | **Subdomains** |
| --- | --- |
| *(1) Good health and optimum nutrition* | • Physical health and capacities. • Mental health and capacities. • Optimal nutritional status and diet |
| *(2) Connectedness, positive values, and contribution to society* | • Connectedness: Is part of positive social and cultural networks and has positive, meaningful relationships with others, including family, peers, and, where relevant, teachers and employers. • Valued and respected by others and accepted as part of the community. • Attitudes: Responsible, caring, and has respect for others. Has a sense of ethics, integrity, and morality. • Interpersonal skills: Empathy, friendship skills, and sensitivity. • Activity: Socially, culturally, and civically active. • Change and development: Equipped to contribute to change and development in their own lives and/or in their communities. |
| *(3) Safety and a supportive environment* | • Safety: Emotional and physical safety. • Material conditions in the physical environment are met. • Equity: Treated fairly and have an equal chance in life. • Equality: Equal distribution of power, resources, rights, and opportunities for all. • Nondiscrimination. • Privacy. • Responsive: Enriching the opportunities available to the adolescent. |
| *(4) Learning, competence, education, skills, and employability* | • Learning: Has the commitment to, and motivation for, continual learning. • Education. • Resources, life skills, and competencies: Has the necessary cognitive, social, creative, and emotional resources, skills (life/decision-making) and competencies to thrive, including knowing their rights and how to claim them, and how to plan and make choices. • Skills: Acquisition of technical, vocational, business, and creative skills to be able to take advantage of current or future economic, cultural, and social opportunities. • Employability. • Confidence that they can do things well. |
| *(5) Agency and resilience* | • Agency: Has self-esteem, a sense of agency and of being empowered to make meaningful choices and to influence their social, political, and material environment and has the capacity for self-expression and self-direction appropriate to their evolving capacities and stage of development. • Identity: Feels comfortable in their own self and with their identity(s), including their physical, cultural, social, sexual, and gender identity. • Purpose: Has a sense of purpose, desire to succeed, and optimism about the future. • Resilience: Equipped to handle adversities both now and in the future, in a way that is appropriate to their evolving capacities and stage of development. • Fulfilment: Feels that they are fulfilling their potential now and that they will be able to do so in the future. |

^1^Ross DA, Hinton R, Melles-Brewer M, et al. Adolescent Well-Being: A Definition and Conceptual Framework. J Adolesc Health. 2020;67(4):472-476.

**Supplemental Table 3.** Demographics and Characteristics of 933 Parent Participants

| **Characteristic** | **Parents (>18 y)**  **Value, No. (%)**  **N=933** |
| --- | --- |
| Sex  Male  Female  Prefer not to answer | 447 (47.9)  486 (52.1)  0 (0.0) |
| Gender  Woman  Man  Non-binary  Two-Spirit  Prefer not to answer | 485 (52.0)  415 (44.5)  6 (0.7)  1 (0.1)  26 (2.8) |
| Age, years  11-14  15-18  19-24  25-34  35-44  45-54  55-64  65-74  ≥75 | 0 (0.0)  0 (0.0)  35 (3.8)  88 (9.4)  349 (37.4)  335 (35.9)  113 (12.1)  12 (1.3)  1 (0.1) |
| Disability  Yes – visible  Yes – invisible  No | 38 (4.1)  83 (8.9)  806 (86.4) |
| Ethnicity  Black, Indigenous, and People of color  White  Prefer to self-describe | 266 (28.5)  654 (70.1)  13 (1.4) |
| Geographic location  Atlantic (BF, NB, NS, PEI)  Central (QC, ON)  Prairies (MB, SK, AB)  West coast (BC) | 41 (4.4)  654 (70.1)  139 (14.9)  99 (10.6) |
| Size of household  <4  ≥5 | 698 (74.8)  232 (24.9) |
| Canadian residence, years  <1  1-4  5-9  10-19  ≥20 | 6 (0.6)  37 (4.0)  131 (14.0)  80 (8.6)  678 (72.7) |
| Self-rated COVID-19 knowledge  Very poor  Poor  Average  Good  Very good | 63 (6.8)  166 (17.8)  198 (21.2)  256 (27.4)  249 (26.7) |
| Previously diagnosed with COVID-19  Yes  No | 293 (31.4)  640 (68.6) |
| Job loss during COVID-19 pandemic  Yes  No  Not applicable | 97 (10.4)  703 (73.4)  133 (14.3) |
| Social media use per day, hours  None  <1  1-3  4-6  >6 | 76 (8.2)  291 (21.3)  356 (38.2)  109 (11.7)  101 (10.8) |

Abbreviations: AB, Alberta; BC, British Columbia; MB, Manitoba; N/A, Not Asked; NB New Brunswick; NS, Nova Scotia; ON, Ontario; PEI, Prince Edward Island; QC, Quebec; SK, Saskatchewan

**Supplemental Table 4.** Unadjusted Multivariate Analyses for Child (11-14 years) Mental Health Symptoms

| **Characteristic** | **Children (11-14 y), N=483** | | |
| --- | --- | --- | --- |
|  | **Mood**  **OR (95% CI)** | **Anxiety**  **OR (95% CI)** | **Irritability**  **OR (95% CI)** |
| Physical health^1^ | 1.19 (0.80-1.79) | 1.22 (0.81-1.82) | 1.01 (0.66-1.53) |
| Mental health^1^ | 1.08 (0.73-1.62) | 1.23 (0.83-1.83) | 1.25 (0.84-1.88) |
| Nutrition^1^ | 0.99 (0.66-1.47) | 1.08 (0.72-1.61) | 0.84 (0.56-1.27) |
| Physical health support^1^ | 1.20 (0.80-1.79) | 1.31 (0.88-1.95) | 1.02 (0.67-1.54) |
| Mental health support^1^ | 1.32 (0.88-1.98) | 1.46 (0.97-2.18) | 1.05 (0.69-1.59) |
| School closure stress^2^ | 1.70 (1.19-2.44) | 2.06 (1.45-2.95) | 1.69 (1.18-2.43) |
| Familial relations^3^ | 4.13 (2.56-6.72) | 4.27 (2.71-6.78) | 2.83 (1.76-4.56) |
| Friend relations^3^ | 2.39 (1.60-3.57) | 2.68 (1.81-3.99) | 2.32 (1.56-3.46) |
| Sleep quantity^4^ | 1.31 (0.93-1.84) | 1.24 (0.89-1.74) | 1.51 (1.07-2.15) |
| Exercise quantity^5^ | 1.62 (1.14-2.30) | 1.30 (0.92-1.83) | 1.15 (0.81-1.63) |
| Social media quantity^6^ | 1.07 (0.70-1.64) | 0.77 (0.51-1.16) | 0.99 (0.65-1.51) |

^1^Disagree versus Neutral or Agree [Prior to the COVID-19 pandemic, I had good…]

^2^Extremely/Very versus Moderately/Slightly/Not at all [How stressful have school closures been for you?]

^3^Worse versus About the same or better [How has the quality of your relations changed?]

^4^<8 hours versus 8+ hours (per night) [During the past two weeks, on average, how many hours did you sleep on weekdays?]

^5^<3 days versus 3+ days (per week) [During the past two weeks, how many days per week did you exercise for at least 30 minutes?]

^6^4+ hours versus <4 hours (per day) [During the past two weeks, how much time did you spend using social media?]

Abbreviations: 95% CI, 95% Confidence Interval

Shaded cells are statistically significant.

**Supplemental Table 5.** Unadjusted Multivariate Analyses for Youth (15-18 years) Mental Health Symptoms

| **Characteristic** | **Youth (15-18 y), N=450** | | |
| --- | --- | --- | --- |
|  | **Mood**  **OR (95% CI)** | **Anxiety**  **OR (95% CI)** | **Irritability**  **OR (95% CI)** |
| Physical health^1^ | 1.26 (0.83-1.92) | 1.54 (1.02-2.34) | 1.70 (1.12-2.60) |
| Mental health^1^ | 1.56 (1.04-2.35) | 2.01 (1.34-3.02) | 1.81 (1.20-2.71) |
| Nutrition^1^ | 1.53 (0.98-2.39) | 1.72 (1.11-2.65) | 1.97 (1.26-3.07) |
| Physical health support^1^ | 1.32 (0.86-2.02) | 1.33 (0.87-2.02) | 1.57 (1.03-2.39) |
| Mental health support^1^ | 1.54 (1.01-2.34) | 1.67 (1.11-2.52) | 2.20 (1.45-3.34) |
| School closure stress^2^ | 2.24 (1.53-3.30) | 2.63 (1.81-3.85) | 3.09 (2.11-4.55) |
| Familial relations^3^ | 6.23 (3.75-10.46) | 5.07 (3.06-8.48) | 6.08 (3.67-10.21) |
| Friend relations^3^ | 3.35 (2.14-5.27) | 2.66 (1.73-4.11) | 4.05 (2.60-6.33) |
| Sleep quantity^4^ | 1.69 (1.14-2.490) | 1.47 (1.01-2.14) | 1.42 (0.97-2.09) |
| Exercise quantity^5^ | 1.66 (1.16-2.40) | 1.50 (1.05-2.14) | 1.98 (1.38-2.85) |
| Social media quantity^6^ | 1.37 (0.92-2.05) | 1.57 (1.06-2.33) | 1.63 (1.10-2.43) |

^1^Disagree versus Neutral or Agree [Prior to the COVID-19 pandemic, I had good…]

^2^Extremely/Very versus Moderately/Slightly/Not at all [How stressful have school closures been for you?]

^3^Worse versus About the same or better [How has the quality of your relations changed?]

^4^<8 hours versus 8+ hours (per night) [During the past two weeks, on average, how many hours did you sleep on weekdays?]

^5^<3 days versus 3+ days (per week) [During the past two weeks, how many days per week did you exercise for at least 30 minutes?]

^6^4+ hours versus <4 hours (per day) [During the past two weeks, how much time did you spend using social media?]

Abbreviations: 95% CI, 95% Confidence Interval

Shaded cells are statistically significant.
